# Bacteria Etiological Agents Causing Lower Respiratory Tract Infections and Their Resistance Patterns

**DOI:** 10.7508/ibj.2015.04.008

**Published:** 2015-10

**Authors:** Salman Khan, Singh Priti, Sachan Ankit

**Affiliations:** 1 *Dept. of Microbiology, Nepalgunj Medical College, Nepal; *; 2 *Dept. of Biochemistry, Nepalgunj Medical College, Nepal;*; 3 *Dept. of Microbiology, Santosh Medical College, India*

**Keywords:** Bacterial infections, Antimicrobial drug resistance, Respiratory system, Nepal

## Abstract

**Background:**

Lower respiratory tract infections (LTRIs) are among the most common infectious diseases with potential life-threatening complications.

**Methods:**

The study consisted of 426 patients with suspected LTRIs from mid and far western region of Nepal between September 2011 and July 2014. The specimens were collected and processed according to the standard microbiological methods at the Central Laboratory of Microbiology of Nepalgunj Medical College, Nepal.

**Results:**

Among the isolated Gram-positive organisms, *Streptococcus pneumonia* (n = 30, 51.7%) was the most predominant pathogen, followed by *Staphylococcus aureus *(n = 28, 48.3%). Among the isolated Gram-negative organisms, *Pseudomonas aeruginosa* (n = 71, 35.32%) was the most predominant pathogen, followed by *Haemophilus influenzae *(n = 68, 33.83%), *Klebsiella pneumonia* (n = 36, 17.19%), and *Escherichia coli* (n = 26, 12.94%). The pattern of resistance varied regarding the bacteria species, and there were multi-resistant isolates. Also, a significant difference (*P* < 0.05) was observed between males and females for each type of bacterial species. Among 259 isolates, 86 (33.20%) were from children aged 1-10 years, which were statistically significant (*P* < 0.05) compared to the other age groups.

**Conclusions:**

*P. aeruginosa* and *H. influenzae *(Gram-negative) and *S. pnemoniae *(Gram-positive) were the most common bacterial isolates recovered from LTRIs. Age group of 1-10 years old was at a higher risk. Many isolates showed appreciable levels of antibiotic resistance due to antibiotic abuse. There is a need to increase surveillance and develop better strategies to curb the increasing prevalence of LRTI in this region of Nepal.

## INTRODUCTION

Respiratory tract infections are common and perhaps the most frequently reported infections of human being. These infections are traditionally divided into upper respiratory tract infections and lower respiratory tract infections (LRTIs). Since these infections are mostly mild, transient-lasting and sometimes self-limiting, many infected individuals tend to disregard them [1]. Of total 3,941,000 deaths in the world, respiratory tract infection accounts for 34.60% deaths in the South-East Region [2]. In addition, LRTI is one of the leading causes of morbidity and mortality worldwide [3]. 

 In developing countries, the situation is more complicated, and management is often difficult due to the problem associated with the identification of the etiological agents and the administration of an appropriate treatment in cases requiring antibiotic therapy [4]. LRTI is not a single disease but a group of specific infection with different epidemiologies, pathogeneses, clinical presentations, and outcomes. The etiology and symptomatology of respiratory diseases vary with age, gender, season, the type of population at risk, and other factors. LRTIs are frequently the first infection to occur after birth and pneumonia is too often the final illness to occur before death [5]. The etiological agents of LRTIs cannot be determined clinically and differ from area to area [6]. Gram-positive bacteria such as *Staphylococcus aureus*, *Streptococcus pneumonia*, etc. as well as Gram-negative bacteria such as *Haemophilus influenza*e, *Pseudomonas*, *Acinetobacter*, and* Klebsiella* species are recovered from LRTIs [6, 7]. 

Monitoring the antimicrobial resistance patterns of the etiological agents is needed not only to guide the clinician when managing cases requiring antibiotic therapy but also to surveil the trend of these infections. Bacteria are known to cause primary infection or super infection, and in most cases, they require targeted therapy. Cases of respiratory tract infections respond to antibiotics treatment though antibiotics abuse since respiratory tract infection is widespread particularly in developing countries and often might lead to resistance [8]. Antibiotic resistance patterns reported for respiratory tract bacteria in other countries, including nearby countries to Nepal, are variables depending on the organism and the drug investigated [9, 10]. However, little is known about the prevalence of microbial agents causing LRTIs in Nepal. Age, gender, and season have been indicated to affect the prevalence of LRTIs [7]. This little knowledge of prevalence of LRTIs warrants for frequent observation on the change in the pattern of antibiogram for these organisms. To our best knowledge, no study has been carried out regarding the bacteria etiological agents causing LRTIs and their resistance patterns in mid and far western region of Nepal. Therefore, this study was conducted to determine the microbial agents of LRTIs and their antibiotic resistance patterns among the patients in mid and far western region of Nepal.

## MATERIAL AND METHODS

A total of 426 patients of various chronic lung diseases from mid and far western region of Nepal were attended in the current study between September 2011 and July 2014. The specimens were collected from LRTI patients representing specimens, viz, sputum, ET secretion, and bronchial washings. The specimens were processed at the central Laboratory of Microbiology for culture and sensitivity which also met the criteria as recommended by American Society for Microbiology were included in the study [11]. Information regarding the duration of hospital stay and antibiotic history was also taken into consideration whenever necessary during the processing of specimens. 

***Specimen culture.*** The sputum was decontaminated by the standard alkaline decontamination method [12]. The digested sputum samples were cultured on Chocolate agar, sheep blood agar (5%), and MacConkey agar (Oxoid, UK) plates. On the Chocolate agar, bacitracin (10 units) and optochin disks (5 µg) (Oxoid, UK) were placed at primary and secondary inoculation to screen *H. influenzae* (type B) and Streptococcus *pnemoniae*, respectively. The Chocolate agar plates were incubated in a incubator (5-10% CO_2_) at 37ºC for 24-48 hours while blood agar and MacConkey agar were incubated in an aerobic atmosphere at 37ºC for 24 hours. Suspicious colonies were then subcultured on suitable solid culture media for purification and thereafter preserved on appropriate agar slants and stored in a refrigerator (4ºC) for subsequent analysis. 

***Identification of isolated organisms.*** The identification of significant isolates were carried out using the standard microbiological techniques, which involved morphological study of colonies, Gram staining reactions, and a battery of biochemical tests as required [13]. A colony count of ≥10^4^ CFU/ml was considered to be significant for bronchial washing [14] while for other specimens, ≥10^5^ CFU/ml was suggestive for infection [15]. The API20E kit (Biomerieux, France) was used for the final confirmation of the isolates following manufacture's instruction.

***Antibiotic susceptibility testing. ***Antimicrobial sensitivity testing was determined by the Kirby-Bauer disc diffusion method [16] on Mueller Hinton agar plates (HiMedia Lab. Pvt. Ltd., Mumbai, India) using the following antimicrobial agents: ampicillin, gentamicin, cefuroxime, chloramphenicol, cipro-floxacin, co-trimoxazole, erythromycin, ceftriaxone, penicillin, ceftazidime, amikacin, aztreonam, cefepime, cefoperazone-sulbactam, piperacillin-tazobactam, ticarcillin-clavulanic acid, and imipenem (HiMedia Lab. Pvt. Ltd., Mumbai, India). The plates were incubated at 37ºC for 24 h, and the diameters of zone of inhibition were compared with those of the reference isolates (*E. coli*, ATCC 25922; *Pseudomonas aeruginosa*, ATCC 27853; *S. aureus*, ATCC 25923; *H. influenza*e, ATCC49247; *S. pneumonia*, ATCC 49619; *K. pneumonia*, ATCC 700603) to determine the susceptibility or resistance based on Clinical and Laboratory Standards Institute guidelines, Wayne, Pennsylvania. [15]. 

***Statistical analysis. ***Data were analyzed using the Statistical Package for Social Sciences software (version 16, SPSS Inc., Chicago, IL, USA). The association of gender and age groups with the prevalence of bacterial species was assessed using Z-score and Chi-square tests. *P* < 0.05 were considered to be statistically significant.

## RESULTS

In total, 426 LRTI specimens were processed according to the standard microbiological methods. Specimens processed in this study were sputum (n = 144), endotracheal secretion (n = 8), and bronchial washing (n = 42). Of 144 sputum specimens, 82 were further processed, and the remaining 62 were excluded because of oral contamination. Also, 154 specimens indicated no growth/sterile samples. Among the total processed specimens (n = 426), only 210 (49.29% [27.46% male and 21.83% female]) showed a significant growth of the different specimens. ETsecretion also displayed the highest microbial isolation (41%), and 80% (n = 168) growth was monomicrobial; however, the rest (20%) was accounted for the mixed growth. The percentages of Gram-negative (n = 201) and Gram-positive (n = 58) bacteria were 77.61% and 22.39%, respectively. The application of Z-score (double sample portion test) revealed a significant difference between males and females for each type of bacterial species at 1% significance level (Table 1). Among 259 isolates, 86 (33.20%) were from children aged 1-10 years, and this group was statistically significant (*P* < 0.05) compared to the other age groups (Table 2).

**Table 1:** Distribution of bacterial species by gender

**Bacterial species**	**Gender**		
	**Male**	**Female**	**Total no. (%)**
*P. aurigenosa*	40	31	71 (27.41)
*H. influenzae*	33	35	68 (26.25)
*K. pneumoniae*	20	16	36 (13.90)
*E. coli*	13	13	26 (10.04)
*S. pneumoniae*	18	12	30 (11.58)
*S. aureus*	14	14	28 (10.81)
Total no. (%)	138 (53.28)	121 (46.72)	259 (100%)

**Fig. 1:**
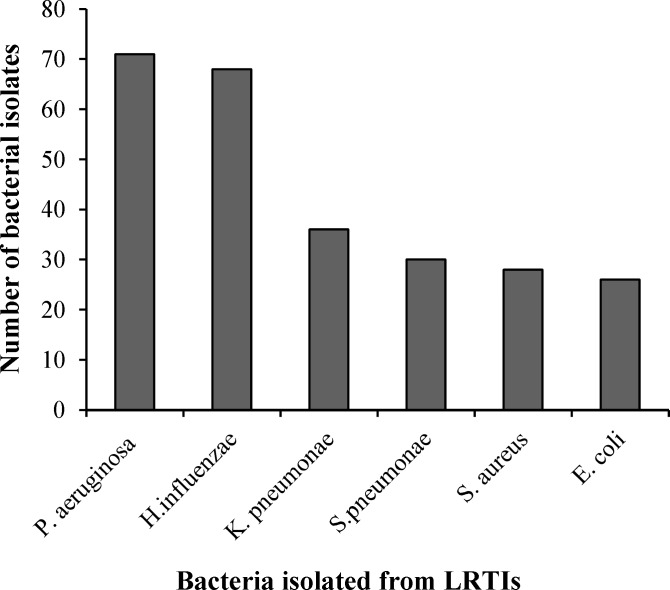
Distribution of total bacterial isolates (n = 259).

***Distribution of bacterial isolates. ***Among the total 259 bacterial isolates,* P. aeruginosa* was found to be the most predominant organism, followed by *H. influenzae*, *K. pnemoniae*, *S. pnemoniae*, *S. aureus*, and *E. coli* (Fig. 1). In addition, among the isolated Gram-positive organisms, *S. pnemoniae* (30, 51.7%) was the most predominant pathogen, followed by *S. aureus* (28, 48.3%), and among the isolated Gram-negative organisms, *P. aeruginosa* (71, 35.32%) was the predominant pathogen, followed by *H. influenzae* (68, 33.83%), *K. pnemoniae* (36, 17.91%), and *E. coli* (26, 12.94%) (Table 3). The resistance pattern of bacteria isolated between September 2011 and July 2014 is shown in Tables 4 and 5. The overall increased resistance was observed for penicillin in* S. aureus *(92.86%), *H. influenzae* (91.18%), *S. pneumonia* (90%), *K. pneumoniae* (88.89%), and *E. coli *(84.62%). These organisms also showed high resistance to co-trimoxazole (82.14%) in *S. aureus*, gentamicin (69.44%) in *K. pneumoniae*, erythromycin (69.23%) in *E. coli*, co-trimoxazole (63.33%) in *S. pneumonia*, and ampicillin (54.41%) in *H. influenzae*. In *P. aeruginosa*, the overall increased resistance was observed for cefepime (56.34%), cefoperazone-sulbactam (52.11%), and gentamicin (47.89%) (Tables 4 and 5).

## DISCUSSION

The primary goal of this study was to ascertain the current prevalence/trend of bacteria causing LTRI among patients in mid and far western region of Nepal. 

**Table 2:** Distribution of bacterial species recovered from different age groups

**Bacterial species**	**Age groups (yr) distribution**							
	**1-10** **no.**	**11-20** **no.**	**21-30** **no.**	**31-40** **no.**	**41-50** **no.**	**51-60** **no.**	**>60** **no.**	**Total** **no (%)**
*P. aurigenosa*	2	5	16	14	16	10	8	71 (42.03)
*H. influenzae*	38	20	8	2	-	-	-	68 (27.54)
*K. pneumoniae*	13	13	4	2	2	2	-	36 (21.74)
*E. coli*	3	1	2	3	9	5	3	26 (8.70)
*S. pneumoniae*	15	10	4	1	-	-	-	30 (11.58)
*S. aureus*	15	8	3	1	1	-	-	28 (10.81)
Total no. (%)	86 (33.20)	57 (22)	37 (14.29)	23 (8.88)	28 (10.81)	17 (6.56)	11 (4.25)	259 (100)

**Table 3:** Distribution of total bacterial isolates according to Gram-negative and Gram-positive bacteria

**Bacteria**	**Bacterial** **species**	**Isolates no.**	**Isolates ** **(%)**
Gram-negative n = 201 (77.61%)	*p. aeruginosa*	71	35.32
	*H. influenzae*	68	33.83
	*K. pneumoniae*	36	17.91
	*E. coli*	26	12.94
Gram-positive n = 58 (22.39%)	*S. pneumoniae*	30	51.7
	*S. aureus*	28	48.3

The antibiogram of the recovered bacterial isolates was also determined in order to ascertain the resistance patterns of these recovered bacterial isolates. 

LRTIs still account for a significant proportion of morbidity and mortality, especially in developing countries [4]. In the current study, the majority of LRTIs were isolated from children aged 1-10 years, and pathogens were recovered from 49.3% of the total specimens. Similarly, 39%, 41%, and 20% of the pathogens were recovered from sputum, ET secretion, and bronchial washings, respectively. One study conducted in the western part of Nepal has recovered respiratory pathogens in 50.4% of samples [17], showing that there is a small decrease in the prevalence of bacterial LRTIs. Moreover, another study was conducted by Mishra *et al. *[18] in Nepal to find out the current trend of the microbial spectrum causing LRTIs. Pathogens were recovered from 44.4% of the total specimens, and similarly 43.7%, 67.2%, and 10% of the pathogens were recovered from sputum, ET secretion, and bronchial washings, respectively.

In a similar manner, prevalence studies performed in China, Turkey, and Iran disclosed that there was a fairly large growth in 53.1%, 59.4%, and 40.0% of the cases, respectively [6, 19, 20]. Use of different types of antibiotics by health professionals has changed the natural history of infectious disease in patients; before the patient comes to Nepalgunj Medical College and Teaching Hospital, Banke, Nepal. The use of antibiotics possibly plays a role in culture negativity. In addition, there is no sensible use of antibiotics in the country, and use of antibiotics without prescription by patients could be another factor contributing to this. Low prevalence of pathogen isolation was also considered in 40-60% of the cases in a study carried out South Asia. The observation that more causative pathogens can be identified in a patient has been demonstrated in several studies. The precise rate of polymicrobial infection is dependent on the laboratory technique, and tested pathogen has been documented to vary from 3% to 40%, and *Chlamydohila pneumonia* appears to be the most common organism of co-infectio [21]. In the current investigation, mono-microbial growth was found in 80% of the cases, whereas 20% were polymicrobial. In another study, monomicrobial growth was found in 91.3% of the cases and 8.7% were polymicrobial [17]. The mixed pathogens were also observed in 11.2% of the samples in the study performed by Ozyilmaz *et al*. [6]. In the present study, Gram-negative bacteria account for 77.61% of all isolated bacteria. The most common isolate was *P. aeruginosa* not only among the Gram-negative bacteria (35, 32%) but also among the total isolates (27, 41%). It is a point of concern that *P. aeruginosa* is the perfect example of opportunistic pathogen of human and is well known for nosocomial infection. The prevalence of *P. aeruginosa* in the present study is higher (35.32%) than that in a study carried out in Manipal Teaching Hospital, Pokhara, Nepal, in which *P. aeruginosa* accounted for 7.5% of LRTI cases [17]. In the current investigation, *H. influenzae* (type B) was 33.83%, which was the highest secondary isolate among the Gram-negative bacteria *H. influenzae *was also common in other studies in Nepal (39.3%) and Turkey [6, 17, 18]. Low prevalence of *H. influenzae* in our study could be due to biofilms formation *in vivo*, which may yield negative cultures [22, 23]. Also, the evidence has indicated that *H. influenzae *is viable inside host cells, including macrophages and respiratory epithelial cells [24]. It is alluring to theorize that *H. influenza* may modify its form of growth under different conditions in the human respiratory tract, responsible for negative sputum cultures. It has been also seen that 34-47% of sputum cultures are negative with confirmed *H. influenzae* pneumonia [25].

**Table 4:** Resistance of the bacterial species isolates to a panel of ten antibiotics

**No.**	**Antimicrobial agent (Concentration [**µg**])**	**Bacterial species**				
		***H. influenzae*** **n = 68** **% (no.)**	***K. pneumoniae*** **n = 36** **% (no.)**	***E. coli*** **n = 26** **% (no.)**	***S. pneumoniae*** **n =30** **% (no.)**	***S. aur*** **eus** **n=28** **% (no.)**
1	Ampicillin (10)	54.41(37)	44.44(16)	42.31(11)	56.67(17)	57.14(16)
2	Gentamycin (10)	16.18(11)	69.44(25)	23.08(6)	13.33(4)	10.71(3)
3	Cefuroxime (30)	8.9 (4)	11.11(4)	19.23(5)	10(3)	28.57(8)
4	Chloramphenicol (30)	32.35(22)	47.22 (17)	42.31(11)	40(12)	25(7)
5	Ciprofloxacin (5)	16.18(11)	22.22(8)	61.54(16)	13.33(4)	10.71(3)
6	Co-trimoxazole (25)	47.06(32)	52.78(19)	57.69(15)	63.33(19)	82.14(23)
7	Erythromycin (15)	22.06(15)	30.56(11)	69.23(18)	33.33(10)	39.29(11)
8	Ceftriaxone (30)	5.88(4)	8.33(3)	11.54(3)	00.00(0)	17.86 (5)
9	Penicillin (10 IU)	91.18(62)	88.89 (32)	84.62(22)	90(27)	92.86(26)
10	Tetracycline (30)	41.18(28)	52.78 (19)	38.46(10)	36.67(11)	60.71(17)

**Table 5:** Resistance of pseudomonas to a panel of ten antibiotics

**No.**	**Antimicrobial agent** **(Concentration [**µg**])**	**Pseudomonas** **n = 71** **% (no.)**
1	Gentamicin (30)	47.89 (34)
2	Ceftazidime (30)	30.99 (22)
3	Amikacin (30)	18.31 (13)
4	Ciprofloxacin (5)	28.17 (20)
5	Aztreonam (50)	30.99 (22)
6	Cefepime (50)	56.34 (40)
7	Cefoperazone-sulbactam (75/30)	52.11 (37)
8	Piperacillin-tazobactam (100/10)	16.90 (12)
9	Ticarcillin-clavulanic acid (75/10)	38.02 (27)
10	Imipenem (10)	0(0)

The respiratory tracts of adults with chronic obstructive disease are colonized by non-type able *H. influenza *responsible for intermittent acute exacerbation [26]. In the present study, 55% of the patients with acute exacerbation of chronic obstructive pulmonary disease showed a growth of *H. influenzae*, which complies with a finding (>50.0%) in Turkey [6]. In this study, Gram-positive bacteria account for 22.39% of all isolated bacteria, and the most common isolate is *S. pneumoniae *(51.7%). *S. pneumoniae* is the organism contributing the highest percentage of Gram-positive as well as Gram-negative isolates. Analysis of 15 published studies from North America over 30 years revealed that the most common causative agent for community-acquired pneumonia (up to 60% cases) was *S. pneumoniae*, which is followed by Gram-negative bacteria, including *H. influenzae*, *K. pneumoniae*,* P. aeruginosa*, *S. aureus*, *Legionella* spp., *Mycoplasma pneumoniae*, and *Chlamydia pneumoniae*, whereas the viruses were least responsible for community-acquired pneumonia [27]. A study in England and Wales also pointed towards a high rate of *S. pneumonia *association for community acquired pneumonia*;* however, *H. influenza* remained the second common isolate [28]. In patients with bacteremic *S. pneumoniae* pneumonia, it has been found that the routine laboratory methods cannot detect the pathogen in 45-50% cases although Gram staining shows large numbers of organisms [29]. According to South Asian Pneumococcal Alliance Project, *S. pneumoniae* is also more common in Nepal [30]. Among 58 Gram-positive isolates besides *S. pneumoniae*, 28 (48.3%) isolates of *S. aureus* were also recovered. A similar result was also found by Liu *et al.* [19] in China. *S. aureus *has emerged as the secondary opportunistic disease, and a prior viral respiratory disease predisposes patient to primary *Staphylococcal pneumonia*. In this study, a considerable number of *S. aureus* were as mixed pathogens. A total of 36 (17.91%) *K. pneumoniae* were isolated, which constituted the third major Gram-negative bacteria. Similar incidence of *Klebsiella* spp. (19.4%) was found from sputum samples by Mishra *et al.* [18] in Nepal. *E. coli *is an uncommon cause of acute LRTIs and in our study, they comprise of 10.04% total cases. The total 26 isolates were found positive for *E. coli*, which was 12.94% among Gram-negative bacteria. This finding corroborates the results obtained (6.9%) by Mishra *et al. *[18].

In the present study, the overall increased resistance shown in Tables 4 and 5 is more or less similar to some recent studies conducted in West African, India, Nigeria, and Nepal [1, 10, 31, 32]. The emergence of fluoroquinolone resistance among LRTIs has now been documented in many countries [8-10, 32]. Nowadays, fluoroqoinolone are substituted by the 3^rd^ generation Cephalosporins, which are frequently used by clinicians. Recent researches have focused on rapidly acquiring resistance to these drugs used in LRTI’s. Resistance to Cephalosporins is plasmid-born and emerging rapidly, which is the factor responsible for reducing the choice of drugs of LRTI treatment. Genetic transfer of drug-resistant gene is not immediate concern for treating clinician but in future, drug resistance will pose a potential problem. Their presence plus the potential for plasmid and mediated quinolone resistance will surely become serious therapeutic problem in future. 

The spectrum of bacteria causing LTRI is still wide in mid and far western region of Nepal. *P. aeruginosa* and *H. influenzae* (Gram-negative) as well as *S. pneumoniae* (Gram-positive) were the common bacteria causing LRTIs in our study. The pattern of resistance also varied according to the bacteria species, and there were multi-resistant isolates. There is great variation in bacterial etiology in different regions and over a specific time in the same regions and populations; therefore, the special surveillance on large population of the children is very important for controlling LRTIs in this age group. Furthermore, the culture of antimicrobial abuse requires to be stopped, and there is a need for continuous surveillance of microbial etiology of LRTI with their resistance pattern.
